# Wearable Sensors Detect Differences between the Sexes in Lower Limb Electromyographic Activity and Pelvis 3D Kinematics during Running

**DOI:** 10.3390/s20226478

**Published:** 2020-11-12

**Authors:** Iván Nacher Moltó, Juan Pardo Albiach, Juan José Amer-Cuenca, Eva Segura-Ortí, Willig Gabriel, Javier Martínez-Gramage

**Affiliations:** 1 Department of Physiotherapy, Universidad Cardenal Herrera-CEU, CEU Universities, 46113 Valencia, Spain; juanjoamer@uchceu.es (J.J.A.-C.); esegura@uchceu.es (E.S.-O.); jmg@uchceu.es (J.M.-G.); 2 Embedded Systems and Artificial Intelligence Group, Universidad Cardenal Herrera-CEU, CEU Universities, 46113 Alfara del Patriarca, Spain; juaparal@uchceu.es; 3 Laboratorio de Investigaciones Biomecánicas, Cátedra de Anatomía Funcional y Biomecánica, Universidad de Buenos Aires, Buenos Aires 1107, Argentina; gwillig@fmed.uba.ar

**Keywords:** running, kinematics, surface electromyography, wearables

## Abstract

Each year, 50% of runners suffer from injuries. Consequently, more studies are being published about running biomechanics; these studies identify factors that can help prevent injuries. Scientific evidence suggests that recreational runners should use personalized biomechanical training plans, not only to improve their performance, but also to prevent injuries caused by the inability of amateur athletes to tolerate increased loads, and/or because of poor form. This study provides an overview of the different normative patterns of lower limb muscle activation and articular ranges of the pelvis during running, at self-selected speeds, in men and women. Methods: 38 healthy runners aged 18 to 49 years were included in this work. We examined eight muscles by applying two wearable superficial electromyography sensors and an inertial sensor for three-dimensional (3D) pelvis kinematics. Results: the largest differences were obtained for gluteus maximus activation in the first double float phase (*p* = 0.013) and second stance phase (*p* = 0.003), as well as in the gluteus medius in the second stance phase (*p* = 0.028). In both cases, the activation distribution was more homogeneous in men and presented significantly lower values than those obtained for women. In addition, there was a significantly higher percentage of total vastus medialis activation in women throughout the running cycle with the median (25th–75th percentile) for women being 12.50% (9.25–14) and 10% (9–12) for men. Women also had a greater range of pelvis rotation during running at self-selected speeds (*p* = 0.011). Conclusions: understanding the differences between men and women, in terms of muscle activation and pelvic kinematic values, could be especially useful to allow health professionals detect athletes who may be at risk of injury.

## 1. Introduction

Recreational running is becoming an increasingly popular pastime [1], with approximately 15% and 70% of amateur athletes currently engaging in this activity in the United Kingdom and the United States, respectively [2,3]. Various studies have shown that 50% of runners suffer an injury each year [4], although there are discrepancies in the literature, due to incidence values that vary from 18.2% to 92.4% [5] and reported prevalence ranging from 46% to 90% among amateur runners [6,7].

In recent years, an increasing number of studies have been published in relation to the biomechanics of running, including factors that could help prevent and treat injuries in runners [8,9,10,11]. Running is a popular recreational activity, but a lack of adequate training in correct running techniques may account for the reported increase in injuries among these athletes [12]. Thus, in this work, we aimed to provide an overview of the different normative patterns of lower limb muscle activation and pelvic joint ranges during running at self-selected speeds in men and women. We analyzed the biomechanics of running by measuring the activation of the main muscles involved in this activity, as well as the dynamic ranges of joint movement, especially in the pelvis [13].

The choice of a preferred speed could be affected by the level of performance and the intensity of the training habits [14]. It is reasonable to expect that amateur runners, with a higher level of performance, will train at higher intensities and, therefore, select a higher running speed for pleasure and metabolic cost [14,15].

Portable dynamic surface electromyography (sEMG) measurement devices, together with inertial sensor units (IMUs), are currently used for this type of analysis [16,17]. These systems provide information about muscle use intensity and activation time, and reflect the different contraction strategies, neuromuscular control systems, and three-dimensional (3D) pelvic kinematics used during running [18,19,20]. The use of wearable systems for these biomechanical measurements allow the data to be captured under more realistic conditions [21].

Given the intrinsic variability of these biomechanical values, the field still lacks a set of reliable reference values for use when assessing both the status and evolution of injured individuals. Some studies have determined these values based on the dynamic range of the pelvis and level of muscle activation by using sEMG for the main muscles involved in running [21,22,23,24,25,26,27]. One study noted increased hamstring and hip flexor tension in runners caused by excess anteroposterior pelvic movement or tilt [23], while in another, back pain was correlated with limited lower knee range [24].

Many studies, exploring the differences in muscle activation in the stance and swing phases of running, are now available in the literature [19,28,29,30]. However, none have systematically categorized the values for muscle function and pelvic kinematics during the different phases of running. Moreover, running mechanics also differ between the sexes, but the differences in the normative patterns of muscle activation, in different phases of running between male and female amateur runners, has not yet been determined [31].

Measuring and characterizing human movements during activity to evaluate athlete performance, improve technique, and prevent injuries is a crucial part of modern training programs [32]. Collecting these data will increase scientific knowledge of kinematic patterns and the degree of muscle activation in runners. Therefore, the purpose of this study was to establish the differences between the sexes in terms of lower limb sEMG activity and three-dimensional (3D) kinematics of the pelvis during running.

## 2. Materials and Methods

### 2.1. Participants

Healthy participants were recruited, who typically engaged in at least 90 min of continuous running training per week, and who had not suffered any injury in the prior year that could have changed their movement patterns. In addition, we excluded individuals who reported having suffered an orthopedic, neurological, or surgical injury in the prior year that could have affected their movement patterns. We explained the nature of the study to all of the participants and they signed their informed consent to participation prior to the start of the work. The entire study was carried out according to the principles of the Declaration of Helsinki, was approved by the ethics committee at CEU Cardenal Herrera University (reference number: CEI18/137), and was registered as a clinical trial (ClinicalTrials.gov registration №: NCT04221698).

### 2.2. Procedure

In this study, we measured the level of activation in the muscles of the dominant leg as well as the pelvic dynamic range of each participant. We used a treadmill (BH Fitness Columbia Pro 130 cm × 40 cm) to establish standardized conditions under which the kinematic variables of running would be more reproducible. We set the incline to 1° and allowed each participant to select the speed [31,32] at which they regularly trained. The participants used their own shoes and were allowed a 15-min warm-up period in order to adjust to the treadmill. According to protocols used in previous running biomechanics studies [28,33,34,35], the initial speed was progressively increased over 2 min and was then maintained for 3 min while the data were collected.

The dynamic range of the pelvis was assessed using an inertial sensor (BTS G-Sensor 2) with an ergonomic belt at the height of S1 to capture different kinematic and spatiotemporal variables. This IMU comprised a 16-axis triaxial accelerometer with multiple sensitivities (±2, ±4, ±6, ±8, and ±16 g) with a frequency of 4 Hz to 1000 Hz, a triaxial gyroscope with multiple sensitivities (±250, ±500, ±1000, ±2000 o/s), with a frequency oscillating between 4 Hz to 8000 Hz, and a triaxial 13-bit magnetometer (±1200 uT), with a frequency exceeding 100 Hz.

Muscle activation was simultaneously studied by sEMG in eight muscles: the gluteus maximus, gluteus medius, rectus femoris, vastus medialis, biceps femoris, semitendinosus, medial gastrocnemius, and soleus. The skin was prepared according to SENIAM guidelines [36], and then two 20 mm pre-gelled self-adhesive bipolar Ag/AgCl disposable surface electrodes (Infant Electrode, Lessa, Barcelona) were placed on each muscle with a 20 mm interelectrode distance between them. A 10 g wireless probe (41.5 × 24, 8 × 14 mm) was placed on each pair of electrodes to capture the sEMG signal and send the information by Wi-Fi to the capture system (BTS FREEMG 1000, BTS Bioengineering, Milan, Italy) via a signal receiver (Wireless IEEE802.15.4) connected to a computer via USB [37].

**Figure 1:**
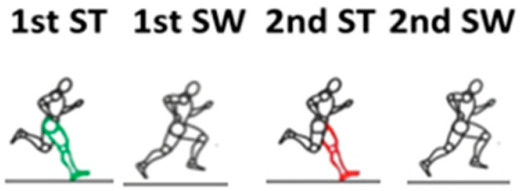
Figure of the running stride cycle sub-cycles: the first stance (1st St), first double float (1st Sw), second stance (2nd St), and second double float (2nd Sw).

The running phases analyzed by sEMG were the percentage of the stride cycle and percentage of each subphase. The start of the stride cycle corresponded to the initial contact and start of the contact of the same foot. The running subphases were: the first stance, first double float, second stance, and second double float (Figure 1). Thus, for the right leg, the first stance occurred from the initial contact of the right foot to the take-off of the right toe. The first double float occurred from the initial float phase of the right foot to the contact of the contralateral foot. This was then followed by the second stance, from the time of initial contact of the left foot to take-off of the left toe, and the second double float from the initial float phase of the left foot until contact of the contralateral foot.

### 2.3. Data Analysis

The EMG signal was recorded simultaneously using a FREEEMG 1000 and EMG Analyzer (BTS Bioengineering, Milan, Italy) that was set to a sampling rate of 1000 Hz per channel, and the signals were band-pass filtered from 20 Hz to 450 Hz. The EMG signals were subsequently full-wave rectified and low pass filtered using a bidirectional, 6th order Butterworth filter, with a cutoff frequency of 5 Hz. The root mean square (RMS) in several subphases was detected. The IMU sensor detected every event performed, initial contact, and toe-off of each foot. Moreover, at the same time, the sEMG signal was recorded, so that the system selected the right and left strides and the different subphases (first stance phase, first float phase, second stance phase, second float phase), as described in Figure 1.

### 2.4. Statistical Analysis

To describe the demographic data of the population sample, descriptive statistics were calculated separately by sex for the participant age, height, weight, and training sessions performed during the chosen week and for the running dynamics data. The data from the study variables were analyzed to check for extreme outlying values using Chauvenet’s criterion, because these may have represented abnormalities in the measurements, musculature, or nerve conduction of the participants.

After testing compliance with the assumptions of normality (Shapiro–Wilk test) and homogeneity of variances (Levene’s test), we decided to use non-parametric methods in our analyses. We used the Wilcoxon rank sum method (based on the Mann–Whitney U test) to compare the sex factor in the biomechanical patterns of pelvis use, muscular activation during the complete running cycle, and the mean activation between men and women at their self-selected speeds. G*Power software was used to calculate the sample size; to detect an effect size of 0.8 with a statistical power of 0.8, we calculated that we would require at least 21 participants in each group. We finally obtained data from 22 men and 16 women, and post-hoc calculations gave us a statistical power of 0.75. RStudio Desktop software (version 1.2.5 for macOS; RStudio Inc., Boston, MA, USA) was used for all of our statistical analyses.

## 3. Results

A total of 48 individuals initially participated in the study, of which eight were considered excluded values because of injury (*n* = 7) or Bluetooth receiver failure (*n* = 1). The demographics of these participants are described in Table 1.

Once the data from the 40 participants included in the trial had been analyzed, 2 participants were excluded because they were considered outliers, leaving a final sample of 38 individuals. Regarding the self-selected speed, the mean for women was 9.22 (±1.59) km/h, and for men it was 10.61 (±1.56) km/h, with this difference being statistically significant. Table 2 shows the mean value and the *p*-value of the difference between the speed and distance between the sexes, calculated using Mann–Whitney U tests.

### 3.1. Kinematics of the Pelvis

**Figure 2:**
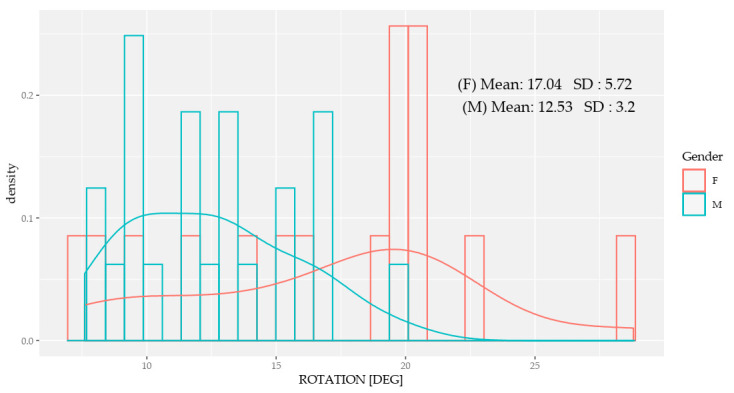
Variation of the rotation between women (F) and men (M), with each bar representing one participant. The lines summarize the distribution of the mean.

Significant differences in the range of pelvic rotation (Figure 2) were observed between the sexes, with female runners presenting a greater range of rotation during running at their self-selected speed, but no significant differences were observed in the tilt or obliquity between the sexes (Table 3).

### 3.2. Mean Running Cycle Muscle Activation

**Figure 3:**
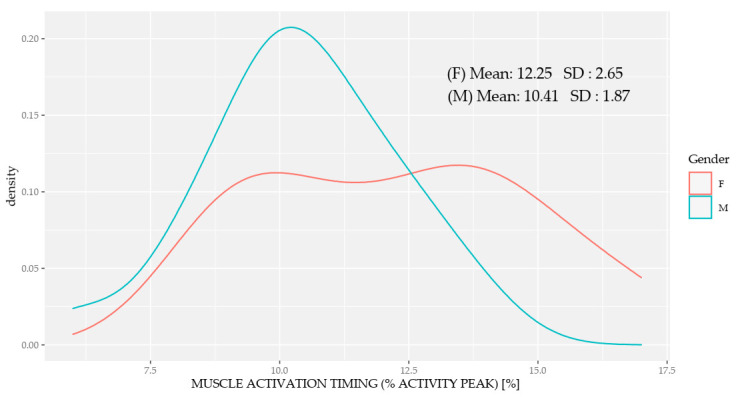
Percentage of the total activation of the vastus medialis during the running cycle distributed between women (F) and men (M). The distribution of the mean and *SD* were more homogeneous in men.

Table 4 shows the statistics for each of the recorded muscles compared by sex for the percentage factor of total muscle activation during each running cycle. The vastus medialis showed a significantly higher percentage of activation in women throughout the running cycle (Figure 3) with a significantly different distribution between the sexes; there was greater muscle activation dispersion in women, indicating increased variability, while the vastus medialis activation homogeneity was reduced in men.

### 3.3. Muscle Activation for Each of the Phases

**Figure 4:**
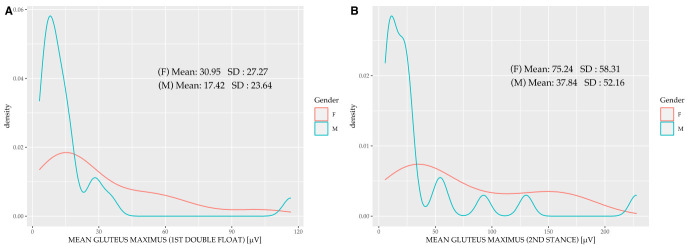
Variation by sex in the gluteus maximus in the first double float (**A**) and second stance (**B**).

**Figure 5:**
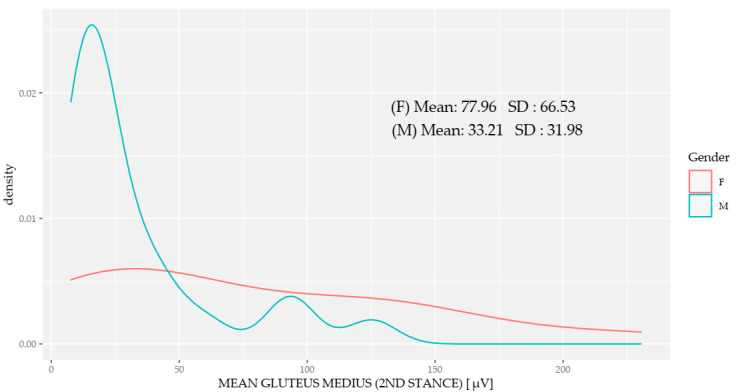
Variation between the sexes in the mean gluteus medius activation during the second stance.

There were significant differences in the muscle activation measurements for each of the phases in each of the main muscles (Table 5). Figure 4 shows the difference in the gluteus maximus muscle activation between women and men running at their self-selected speeds. The distribution of the muscle activation in men was more homogeneous and presented significantly lower values than for women. Figure 5 shows the difference in gluteus medius muscle activation between women and men during the second stance, showing lower homogeneity in women and greater activation than in men.

## 4. Discussion

The main objective of this study was to establish whether there were differences between the sexes in muscular activation or in the 3D kinematics of the pelvis during the entire running cycle and in each of the running phases. We started with the hypothesis that the sex factor could determine the level of muscle activation throughout the running cycle and its component phases. Our results show that there were differences between the sexes, in terms of the total percentage of muscle activation during the entire running cycle in the vastus medialis. In addition, there were differences in the use of this muscle and the gluteus maximus between the sexes in the individual running phases. Moreover, there were sex differences in the rotation of the pelvis. The differences between the sexes in terms of the speed and distance traveled were similar to those previously described in the literature [21].

Similar to the cohorts used in other studies [19,38,39,40], the participants in this work were recruited through random sampling, following established inclusion criteria, from among a population of amateur runners of different ages. We allowed the participants to self-select the speed at which they ran because, in addition to the effects of the age and body mass and body composition factors [17], running speed is directly related to cardiovascular factors, such as individual aerobic threshold and performance [16], and with biomechanical factors, such as stability, flight time, and leg contact time [41]. In this same sense, work by Zamprano et al. [14] and Lussiana et al. [41] indicated that the speed chosen by each participant is related to their energy saving strategy. Thus, imposing a set speed upon runners, rather than allowing them to select the speed at which they are comfortable running, caused lower limb biomechanical changes and produced alterations in the muscle activation pattern and pelvis dynamics. These data are reinforced by those published by Kong et al. [42], which concluded that self-selected velocities would eliminate abnormal kinematic patterns.

In this work, we placed the inertial sensor at the S1 level as a reference to quantify the kinematics of the pelvis. However, we are unable to compare our data with other methodologies, because no previously published work contrasted the kinematic data of the pelvis during running at self-selected speeds, except for the work by Perpiñá et al. [24], who also placed the sensor at level S1. There were no significant differences between the sexes for the tilt range or pelvic obliquity kinematic values obtained. These values coincided with the expected normal values and were not novel. However, we did find that the mean lower pelvic rotation range for women (17.04° ± 5.72°) was significantly higher than the values found for men (12.53° ± 3.2°). Furthermore, the rotational ranges in men were lower than the reference values of 16°–18° provided in studies that dynamically measured the pelvis during running, perhaps because of differences in the speed used [28,43].

When the toes take off during the propulsion phase in running, the pelvis presents its maximum anterior tilt level, slight ipsilateral obliquity to support, and slight external rotation [27,42]. This limits hip flexion and makes rotation the most advantageous mechanism to lengthen the stride. This increased pelvic rotation in women seems to be related to a genetic predisposition towards greater flexibility [44] and a lower capacity for elastic energy storage [45]. All of this is associated with a decrease in the peak vertical forces used by female runners [46]; thus, requiring rotational compensation at every speed. Therefore, women must increase their dynamic range of rotation to increase their hip extension without altering the other kinematic variables and muscle activation factors. This would lead to greater stability and running economy in women due to structural differences in the female pelvis and hips compared to males [22].

We also found different muscle activation responses in the different running phases according to the muscles studied. The gluteus medius is activated in women because they have increased pelvis–hip joint movement and the main function of this muscle is to stabilize these joints. Thus, when the ground reaction force is absorbed in the first part of the first or second stance, the gluteus medius performs more eccentric work in women than in men [39]. In contrast, this muscle causes hip abduction in the first and second take-off phases [47,48]. Therefore, women require increased gluteus medius activation to meet the biomechanical requirements of running, particularly in the second stance. This can lead to the appearance of injuries, either because of a lack of activation or because of fatigue, which are both of primary clinical importance because these factors strongly correlate with the appearance of injuries [49,50,51,52,53].

The gluteus maximus is activated when the foot first contacts the ground and stops hip and trunk flexion in this phase [51]. This muscle also performs trunk extension and strengthens the knee when it is fully extended by acting through the iliotibial tract [54]. Gluteus maximus activity increases during the flight and swing phases because, together with the hamstrings and psoas, it behaves as a hip and knee accelerator during this phase [55]. In agreement with the data from this current study, several other authors also believe that contraction of this muscle at the midpoint of the oscillation phase (between the first double float and second stance) is involved in leg deceleration [56] and may also be related to passive extension of the knee.

We obtained a mean gluteus maximus activation of less than 30.95 μV for men in the first double float phase in this study, which may correspond to a gluteus maximus activation deficit. In contrast, activation of this muscle in female runners in the second stance was below 75.24 μV. Furthermore, the hamstring muscles in this study showed increased activity to control hip flexion when the trunk was flexed, which was causally related to pelvis stabilization [57,58,59]. Maximal medial and lateral hamstring activity during running occurs through eccentric contraction in the middle of the swing phase in order to decelerate the leg just before maximal hip flexion, and immediately after the start of the knee extension [60,61].

The increase in vastus medialis muscle activity we observed in women compared to men (as a percentage of the overall running cycle), as well as during the swing in the first double float and second stance phases, may be because women tend to be less stiff than men. This would reduce their energy storage capacity in the transverse and frontal planes of the trunk and hip muscles [45], thus, decreasing the stability of passive structures and increasing their range of motion, in turn leading to greater stabilization at the muscular level [62,63,64].

Another function attributed to the vastus medialis is stabilization of the patella within the trochlear groove [63,64,65], thus, generating a medializing force vector upon the patella, which would cause its rotation when in extension [66,67,68]. The quadriceps are also active during the swing phase of running, in preparation to receive the weight load [69]. Interestingly, women seem to have increased quadriceps activation when performing sports activities [70], which can substantially contribute to physiologically significant [71,72,73] changes in muscle strength between the sexes [71].

Our data also agreed with previous work showing that vastus medialis activation for hip muscle recruitment differs in women when in positions that are neutral or with a slight medial hip rotation [74,75]. Indeed, Montgomery et al. concluded that contraction of this muscle is required in the swing phase to provide knee extension, thus, stabilizing the patella before the heel strike [73]. In addition, compared to men, we found structural and anatomical differences in the lower limbs of women during running. This reduced normative pattern of vastus medialis activation in women may help them cope with external forces. This is important because it would generate a neuromuscular imbalance between the vastus of the quadriceps, thus, producing a greater risk of injuries, such as patellofemoral pain in female runners [72,76].

To the best of our knowledge, this is the first time normative patterns for the running kinematics of the major muscles and range of motion of the pelvis have been specifically established for each sex. Our results support the stabilizing role the gluteus medius has on the pelvis and knee, as well as the role of the vastus medialis in balancing the patella and controlling the knee valgus during running. The co-contraction of these muscles, together with that of the gluteus maximus and hamstrings, produces adequate motor control. These data could prove useful in clinical settings to prevent the injuries most frequently found in female amateur runners.

One of the limitations of this work may be its sample size (although it was similar to the cohort sizes used in other studies) because it could limit statistical interpretation with the aim of establishing normative data. Furthermore, we did not consider the influence of age, which could have affected the choice of our participants’ running speeds, as well as their running economies [40]. Finally, this study was novel, so the lack of publications about normative muscle activation levels and normative pelvic kinematic patterns limited our ability to compare these data with other work; this makes it harder to understand the true causes of the differences we found between the sexes. Future studies should analyze the differences between healthy individuals and those with certain running injuries in order to analyze their possible origins. This could allow personalized training and prevention plans to be established, and could increase the recovery speed in individuals who already have an injury.

## 5. Conclusions

In conclusion, these differences between the sexes, in terms of muscle activation and pelvic kinematic values, could be especially useful for detecting athletes who may be at risk, allowing healthcare professionals to intervene before possible injuries appear. Here, we found a normative pattern of increased pelvic rotation as well as an increase in gluteus maximus, gluteus medius, and vastus medialis muscle activation in female runners. Further studies will be required to examine whether these differences in pelvic kinematics and muscle activation are related to the injuries commonly experienced by female and male recreational runners.

## Figures and Tables

**Table 1:** Participant characteristics *.

	Value	
	Female	Male
Participants, *n*	16	22
Age, years	27.07 ± 9.16	26.39 ± 6.61
Weight, Kg	58.31 ± 7.06	70.14 ± 8.3
Height, cm	166.3 ± 0.06	177.5 ± 0.07
Weekly number of training sessions	3.93 ± 1.03	4.87 ± 1.14

* Values represented as the mean and standard deviation (*SD*).

**Table 2:** Statistics and significance between sex and the speed and distance variables *.

	Female (avg)	Female *SD*	Male (avg)	Male *SD*	Wilcoxon *p*-Value
Speed (km/h)	9.22	1.59	10.61	1.56	0.009 *
Distance (Km)	0.79	0.13	0.9	0.14	0.02 *

* Significant differences at *p* < 0.05. Speed expressed in kilometers/hour and distance measured in kilometers.

**Table 3:** Differences between men and women in the kinematics of the pelvis during sprinting at a self-selected speed *.

Variable	Mean Men	Mean Women	*p*-Value
Rotation	12.53 (*SD:* 3.2)	17.04 (*SD:* 5.72)	0.011 *
Obliquity	7.57 (*SD:* 1.99)	7.82 (*SD:* 1.61)	0.391
Tilt	7.41 (*SD:* 1.68)	8.51 (*SD:* 2.11)	0.086

* Mean values with their standard deviations (*SD*) are shown. * Statistically significant differences at *p* < 0.05.

**Table 4:** Statistics and significance of the percentage of total muscle activation during the running cycle *.

Muscle	% Activation Women	% Activation Men
Gluteus maximus	12 (11.25–15.50)	12 (11–13)
Gluteus medius	12 (11–13)	11.50 (10.75–13)
Femoral rectus	12 (11–14)	13.50 (12–15.25)
Vastus medial	12.50 (9.25–14) *	10 (9–12) *
Semitendinosus	14 (13–15.75)	13 (11.75–16)
Femoral biceps	14.50 (13.25–17.30)	15.00 (13–15)
Medial gastrocnemius	10.50 (9–12)	11.00 (10–12)
Soleus	10.00 (10–11.75)	12 (11–13.70)

* Percentage value of the median (25th–75th percentile). * Significant differences at *p* < 0.05.

**Table 5:** The *p*-values of the mean in the muscles with significant differences between the sexes in different phases.

Muscle	1st Stance	1st Double Float	2nd Stance	2nd Double Float
Gluteus maximus	*p* = 0.114	*p* = 0.013 *	*p* = 0.003 *	*p* = 0.647
Gluteus medius	*p* = 0.198	*p* = 0.057	*p* = 0.028 *	*p* = 0.584

* Significant differences at *p* < 0.05.
